# *codY* and *pdhA* Expression Is Induced in *Staphylococcus epidermidis* Biofilm and Planktonic Populations With Higher Proportions of Viable but Non-Culturable Cells

**DOI:** 10.3389/fcimb.2021.771666

**Published:** 2021-11-15

**Authors:** Vânia Gaio, Nathalie Lopes, Nuno Cerca, Angela França

**Affiliations:** Laboratory of Research in Biofilms Rosário Oliveira (LIBRO), Centre of Biological Engineering (CEB), University of Minho, Braga, Portugal

**Keywords:** *Staphylococcus epidermidis* infections, VBNC cells induction model, planktonic cells, biofilms, glucose, magnesium, gene expression

## Abstract

*Staphylococcus epidermidis* biofilm cells can enter a physiological state known as viable but non-culturable (VBNC), where, despite being alive, they do not grow in conventional laboratory media. As such, the presence of VBNC cells impacts the diagnosis of *S. epidermidis* biofilm-associated infections. Previous transcriptomics analysis of *S. epidermidis* strain 9142 biofilms with higher proportions of VBNC cells suggested that the genes *pdhA, codY* and *mazEF* could be involved in the induction of the VBNC state. However, it was previously demonstrated that VBNC induction is strain-dependent. To properly assess the role of these genes in VBNC induction, the construction of mutant strains is necessary. Thus, herein, we assessed if VBNC cells could be induced in strain 1457, a strain amenable to genetic manipulation, and if the previously identified genes were involved in the modulation of the VBNC state in this strain. Furthermore, we evaluated the formation of VBNC cells on planktonic cultures. Our results showed that despite being commonly associated with biofilms, the proportion of VBNC cells can be modulated in both biofilm and planktonic cultures and that the expression of *codY* and *pdhA* was upregulated under VBNC inducing conditions in both phenotypes. Overall, our study revealed that the formation of VBNC cells in *S. epidermidis* is independent of the mode of growth and that the genes *codY* and *pdhA* seem to be relevant for the regulation of this physiological condition.

## Introduction

*Staphylococcus epidermidis* is now considered an opportunistic pathogen responsible for many healthcare-associated infections, mainly those related to biofilm formation on indwelling medical devices (Mack et al., 2013). *S. epidermidis* biofilm-associated infections are often recurrent leading to high rates of morbidity (Otto, 2009). The failure of antibiotics to cure these types of infections is primarily associated with the poor capacity to eradicate biofilms, which are known to have bacterial cells with distinct physiological states (Rani et al., 2007), including viable but non-culturable (VBNC) cells (Cerca et al., 2011a; Zhang et al., 2018; Li et al., 2020). Although VBNC cells cannot grow on standard growth media, these cells present a reduced metabolic activity, replication rate and gene transcription (Lleo et al., 2000; Zhang et al., 2018). For this reason, their detection with traditional culture-based methods and, consequently, the diagnosis of *S. epidermidis* biofilm-related infections is hindered (Zandri et al., 2012). Moreover, *S. epidermidis* VBNC cells can be more tolerant to antibiotics (Cerca et al., 2014), limiting the efficacy of current treatment options. As such, the study of the mechanisms underlying the development of this physiological state in *S. epidermidis* is of utmost importance.

An *in vitro* model aimed to induce the formation of VBNC cells in *S. epidermidis* biofilms, developed on strain 9142, demonstrated that the addition of a high concentration of glucose (1%) increased the proportion of VBNC cells, while the addition of magnesium chloride (MgCl_2_, 20 mM) prevented the formation of VBNC cells. This modulation resulted in biofilms with approximately the same number of viable cells but about 1 log difference in the number of culturable cells (Cerca et al., 2011a; Carvalhais et al., 2014). Importantly, although glucose enrichment was associated with medium acidification, it was formerly demonstrated that the prevention of the VBNC state is a pH-independent phenomenon since the addition of MgCl_2_ does not prevent acidification of the medium (Cerca et al., 2011a). The applicability of this model to induce the formation of VBNC cells in *S. epidermidis* biofilms was further analysed in 19 clinical and 24 commensal isolates (Carvalhais et al., 2018). Most of the clinical isolates tested (70%) showed at least a 0.5 log_10_ decrease in culturability, whereas only 33% of the commensal isolates presented a similar reduction, suggesting that VBNC cells induction by glucose and prevention by magnesium chloride is not a universal phenomenon amongst *S. epidermidis* isolates. A possible explanation for this isolate-specific response may be related to transcriptomic changes, for instance, in genes involved in metabolism and oxidative stress (Keren et al., 2011; Carvalhais et al., 2014; Postnikova et al., 2015). Therefore, the analysis of genes whose products could be associated with the regulation of this physiological state is crucial to underpin the mechanisms behind its emergence.

Previously, using an RNA-Sequencing (RNA-Seq) approach, we have detected changes in the transcription of the genes *codY*, *mazE*, *mazF* and *pdhA* in biofilms with a higher proportion of VBNC cells, suggesting that these genes could be linked to the emergence of the VBNC state in *S. epidermidis* strain 9142 (Carvalhais et al., 2014). To confirm this hypothesis, the study of strains lacking the genes of interest is essential. *S. epidermidis* is known to be difficult to be genetically manipulated, with only a few strains known to be amenable (Mack et al., 2001; Winstel et al., 2015; Winstel et al., 2016; Galac et al., 2017), being *S. epidermidis* strain 1457 the most frequently used for mutagenesis studies. Unfortunately, earlier studies aiming to modulate VBNC cells proportions in *S. epidermidis* biofilms did not include such strain. Therefore, to determine if *S. epidermidis* 1457 could be a suitable candidate for the study of the mechanisms behind the formation of VBNC cells, the ability of this strain to form VBNC cells needs to be investigated.

Thus, herein, we tested the induction of VBNC cells in biofilms formed by strain 1457, using the previously optimized *in vitro* VBNC cells induction model, and assessed the expression of the genes *codY*, *mazE*, *mazF* and *pdhA*. Additionally, we investigated the suitability of this model in planktonic cultures, further exploring the potential role of the previously identified genes in the VBNC state mediation in planktonic cells.

## Material and Methods

### Strains and Growth Media

*S. epidermidis* 1457, a strain isolated from a venous catheter-associated infection (Mack et al., 1992) was used for this study, together with the commensal isolate COM040A (skin sample from a healthy volunteer) and the clinical isolate PT11004 (isolated from a bloodstream infection), which were previously shown to accumulate high amounts of VBNC cells (Carvalhais et al., 2018). Additionally, to validate previous RNA-Seq data, strain 9142 was also included. For each experiment, all *S. epidermidis* strains were grown directly from the glycerol stocks (30% glycerol) in tryptic soy broth (TSB, Merck, Darmstadt, Germany) at 37°C and with shaking at 120 rpm (10 mm orbit shaker). The optical density (OD) of overnight suspensions was adjusted, at 640 nm, to 0.25 ± 0.05, corresponding to ≈ 2 × 10^8^ colony-forming units/mL (CFU/mL) (Freitas et al., 2014), to be used as inoculum for all the experiments subsequently described.

### Biofilm Cultures

Biofilms were formed, in 24-well plates (Orange Scientific, Braine-l’Alleud, Belgium), by inoculating 10 µL of overnight suspensions, previously adjusted to OD_640nm_= 0.25 ± 0.05, into 1 mL of TSB supplemented with 0.4% glucose or 0.4% glucose plus 20 mM MgCl_2,_ for further induction and prevention of the VBNC state, respectively (Cerca et al., 2014). The plates were then incubated for 24 h at 37°C and 120 rpm. After that period, the spent media was removed and replaced by fresh TSB supplemented with either 1% glucose (induced VBNC state) or 1% glucose plus 20 mM MgCl_2_ (prevented VBNC state), and grown under the same temperature and agitation conditions for additional 24 h. Thereafter, the biofilm bulk fluid was removed, and the biofilms washed twice with 500 µL of 0.9% NaCl. Finally, biofilms were scraped from the plate bottom and suspended in 1 mL of the same saline solution.

### Planktonic Cultures

For the analysis of VBNC cells formation in planktonic cells, 24 h and 48 h cultures, grown in 10 mL erlenmeyers, were evaluated. In the case of 24 h planktonic assays, a 1:100 dilution of the overnight growth was performed in TSB supplemented with 1% glucose (induction of the VBNC state) or 1% glucose plus 20 mM of MgCl_2_ (prevention of the VBNC state) and incubated at 37°C and 120 rpm for 24 h. The second approach (48 h growth) aimed to mimic the conditions used for biofilms growth. This consisted of pre-growing bacteria with TSB supplemented with 0.4% glucose (plus 20 mM of MgCl_2_ for prevented VBNC condition) for 24 h, followed by centrifugation of the cells (16.000 g, 10 min, 4°C) and replacement of the spent medium with fresh TSB supplemented with 1% glucose or 1% glucose plus 20 mM MgCl_2_. The suspensions were then grown for additional 24 h under the same temperature and agitation conditions.

### Assessment of VBNC State Induction in Both Biofilm and Planktonic Cultures

At the selected time points, bacterial cells from either biofilms or planktonic cultures were collected and sonicated for 10 s at 33% amplitude (Ultrasonic Processor Model CP750, Cole-Parmer, IL, USA) to dissociate cells clusters and create a homogeneous biofilms cells suspension. Importantly, the selected sonication cycle has no significant effect on cells viability, as previously determined by CFU counting and propidium iodide incorporation (Freitas et al., 2014). The quantification of the total amount of suspended cells was performed by OD_640nm_ measurements, as previously shown (Freitas et al., 2014). Viable cells were quantified by flow cytometry using SYBR Green (1:80000)/propidium iodide (20 µg/mL) staining as previously optimized (Cerca et al., 2011b). Samples were acquired in an EC800™ flow cytometer (Sony Biotech, CA, USA), with a flow rate of 10 µL/min and a total of 100000 events were acquired for each sample. Data analysis was performed using FCS Express 6 and considering the populations SYBR^+^/PI^-^ (live cells) and SYBR^+^/PI^+^ (live cells somewhat permeable to PI), and excluding SYBR^-^/PI^+^ (dead cells). Finally, the number of culturable cells was determined by CFU counting. Briefly, serial dilutions were performed in 0.9% NaCl and 5 µL of each dilution were plated on TSA plates. Plates were incubated at 37°C for at least 16 h. The analysis of the proportion of VBNC cells in both biofilm and planktonic cultures was determined as the ratio (%) between the values obtained for induced and prevented conditions (IND/PRE), in terms of culturability (CFU) or OD.

### Gene Expression Quantification by Quantitative PCR (qPCR)

#### RNA Extraction

For total RNA isolation from biofilm cells, the bulk fluid of the biofilm culture was discarded, the biofilms washed twice with 0.9% NaCl and then suspended in the same solution by scraping the cells from the plate bottom. All the procedure was performed on ice. Three independent biofilms were pooled to reduce biological variability (Sousa et al., 2014) and immediately centrifuged at 16000 g for 10 min at 4°C. For RNA isolation from planktonic cultures, 1 mL of culture was collected and immediately centrifuged at 16000 g for 10 min at 4°C. Of note, bacterial cells were suspended in 0.9% NaCl before RNA isolation since we have previously determined that mRNA quantification was similar to when using RNA preserving solutions if processed immediately (**Supplementary Figure 1**). The extraction of RNA from both suspensions was then performed using the kit ExtractMe RNA Bacteria & Yeast (Blirt S.A., Gdansk, Poland) as previously optimized (França et al., 2012). In brief, bacterial pellets were suspended in 600 µL of RYBL buffer, transferred into 2 mL tubes containing 0.5 g of acid-washed silica beads (150–212 mm) (Sigma-Aldrich, USA) and the cells were lysed using a BeadBug 6 Microtube Homogenizer (Benchmark Scientific, NJ, USA) for 35 s at ~4.5 rpm. Subsequently, samples were incubated on ice for 5 min and the cell disruption and cooling steps repeated three more times. Afterwards, samples were centrifuged at 16000 g for 1 min at 4°C, the supernatants transferred into 2 mL RNase-free tubes and mixed with an equal volume of 70% ethanol. The subsequent steps were performed according to the manufacturer’s instructions. RNA samples were then treated with DNase I (Thermo Fisher Scientific Inc, MA, USA) to degrade contaminating genomic DNA. RNA concentration and purity (A_260/A280_ and A_260/A230_) were determined by NanoDrop One (Thermo Fisher Scientific Inc) and RNA integrity was inferred by visualization of the 23S/16S rRNA banding pattern using a 1% non-denaturing agarose gel.

#### Complementary (c) DNA Synthesis

Total RNA concentration was adjusted to 250 ng in all samples and then reverse transcribed, in a 10 µL reaction volume, using RevertAid H minus Reverse Transcriptase enzyme (M-Mulv RT, Thermo Fisher Scientific, Inc.) and random primers (Bioron, Römerberg, Germany) as priming strategy. The synthesis was performed following the manufacturer’s instructions. A control lacking the reverse transcriptase enzyme (no-RT control) was prepared to later determine the level of genomic DNA contamination.

#### qPCR

The primers used for qPCR were designed with the support of Primer3 software (Untergasser et al., 2012) and using *S. epidermidis* RP62A (for *16S* rRNA primers) or 1457 complete genome as a template (NCBI accession no. CP000029.1 and CP020463.1, respectively) (**Supplementary Table 1**). qPCR analysis was prepared in a 10 µL reaction containing 2 µL of diluted cDNA or no-RT control (1:400), 5 µL of Xpert Fast SYBR Mastermix (GRiSP, Lda., Porto, Portugal), 0.5 µL of each forward and reverse primers (0.5 µM per reaction), and 2 µL of water. qPCR run was performed in a CFX96 (Bio-Rad, CA, USA), with the following cycle parameters: 95°C for 2 min, and 40 cycles of 95°C for 5 s, 60°C for 30 s. A no-template control was included to assess reagent contamination and a melting curve analysis was performed to ensure the absence of unspecific products and primer dimers. Reaction efficiency was assessed at 60°C by performing 10-fold dilution series of the cDNA samples and determined from the slope of a standard curve. The expression of the genes tested was normalised to the expression of the reference genes *16S rRNA* and *gyrB* using a variation of the LivaK method, according to Eq. (1), where E stands for the reaction efficiency. To simplify the analysis between strains and conditions, the results are represented as the ratio of TSB_1%+Mg_/TSB_1%G_ (Fold-change IND/PRE).


Eq. (1) $$E^{\Delta Ct}=E^{Ct_{geometric\;mean\;of\;16S\;rRNA/gyrB}-Ct_{target\;gene}}$$

### Statistical Analysis

Statistical differences between conditions were determined using either unpaired T-test with Welch’s correction or One-way ANOVA with Tukey’s multiple comparisons test, using GraphPad Prism version 7 (Trial version, CA, USA). A *p*-value less than 0.05 was considered significant. At least three independent experiments were performed for each assay presented.

## Results

### Validation of VBNC State Induction Model in *S. epidermidis* 1457 Biofilms

**Figure 1:**
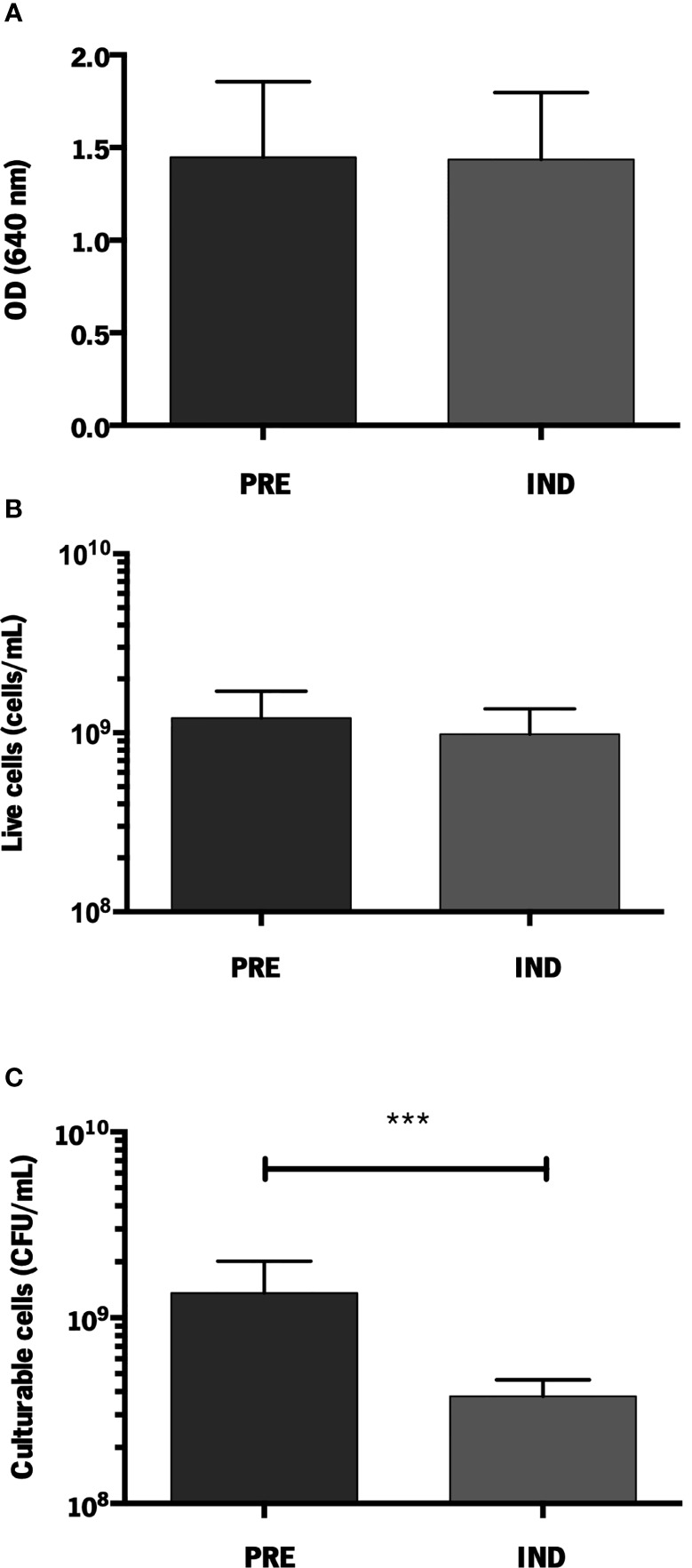
Quantification of *S. epidermidis* 1457 biofilms formed under VBNC conditions. **(A)** Total amount of cells (OD_640 nm_); **(B)** Live cells (cells/mL) and **(C)** Culturable cells (CFU/mL). The results are displayed as the mean + standard deviation of at least six independent experiments. ***p < 0.001 (One-way ANOVA). PRE, prevented; IND, induced.

To assess if the previously described VBNC induction model could be used in strain 1457, we first compared the total amount of cells (assessed by optical density), the number of living cells (assessed by flow cytometry) and the number of culturable cells (assessed by CFU quantification). For biofilms with an equivalent amount of total and live cells, the induction of the VBNC state resulted in a significantly lower number of cultivable cells, confirming that the formation of VBNC cells in stain 1457 can be induced (**Figure 1**). Additionally, no differences were found regarding the pH of the media (data not shown), confirming that VBNC state prevention with MgCl_2_ did not affect the pH of the culture, as previously shown (Cerca et al., 2011a).

To better correlate our data with previously published results, we compared the level of VBNC cells in strain 1457 with the reference strains COM040A and PT11004, whose significant ability to enter into a VBNC state was formerly confirmed (Carvalhais et al., 2018). As we showed above that flow cytometry and OD measurements yielded similar results, VBNC quantification was further analysed by comparing the total amount of cells (OD) and total culturable cells (CFU). Although the 70% decrease in culturability in strain 1457 was lower than the one found in the reference isolates (≈ 90%) (**Supplementary Figure 2**), it is still within the range previously considered relevant in the context of VBNC state induction (Carvalhais et al., 2018).

### Validation of RNA-Seq Results by qPCR

**Figure 2:**
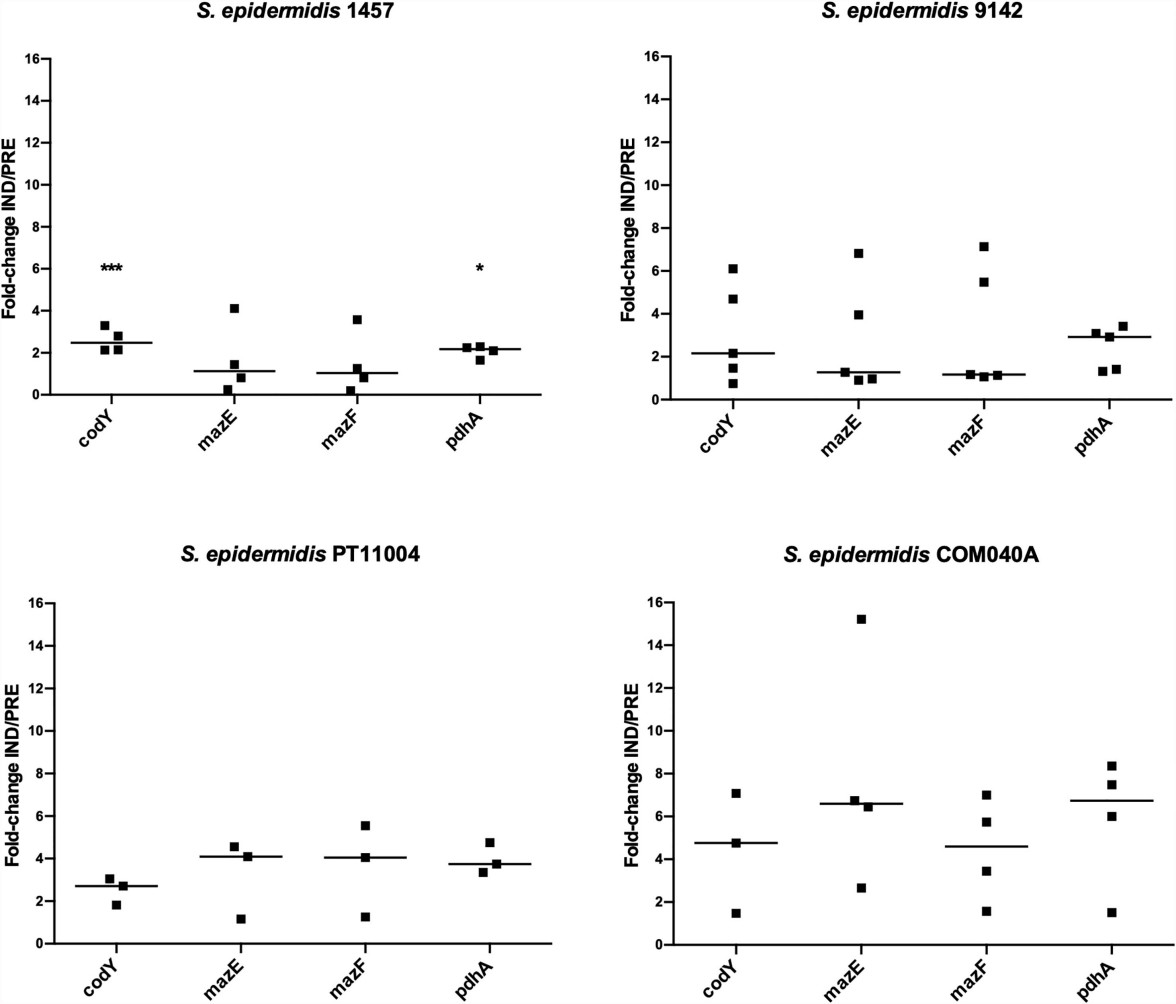
Expression of the genes of interest in 48 h-old biofilms populations of *S. epidermidis* 1457 grown under induced (IND) and prevented (PRE) VBNC conditions. Data is presented as the fold-change between the IND and the PRE VBNC conditions (IND/PRE). Data is represented as the fold-change of the individual assays, where the horizontal lines represent the median of at least three independent experiments. *p < 0.05, ***p < 0.001 (Unpaired Welch’s T-test).

Aiming to determine possible candidates for future mutagenesis studies, the expression of the genes highlighted in a former RNA-Seq analysis of *S. epidermidis* 9142 biofilms under inducing VBNC conditions was validated by qPCR (**Figure 2**). The first step was to compare the results obtained by RNA-Seq and qPCR for biofilms of strain 9142. Although a higher fold-change was observed in qPCR results, the ratios were not significantly different from the ones obtained by RNA-Seq, confirming the results previously obtained (*codY*, 1.5 ± 0.4; *mazE*, not applicable; *mazF*, 1.0 ± 0.6 and *pdhA*, 1.7 ± 0.2) (Carvalhais et al., 2014). Secondly, we assessed the expression of the selected genes in biofilms of the reference isolates, as well as in strain 1457. Not surprisingly, strain-to-strain variability was observed. Nevertheless, qPCR data confirmed that all tested genes were upregulated under VBNC inducing conditions in the reference isolates. Interestingly, although in strain 1457 the expression of the genes *codY* and *pdhA* was significantly increased in the induced VBNC state, the expression of the *mazEF* complex was not significantly affected.

### Applicability of the VBNC State Induction Model in Planktonic Populations

**Figure 3:**
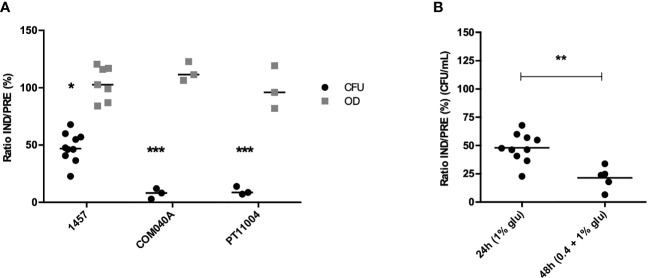
Culturability (CFU/mL) and total amount of cells (OD_640nm_) of **(A)** *S. epidermidis* 1457, COM040A and PT11004 24 h-old planktonic suspensions grown under induced (IND) and prevented (PRE) VBNC conditions. Data is represented as the ratio (%) of the culturability (CFU) or OD between cells in the IND or PRE VBNC state; and **(B)** *S. epidermidis* 1457 planktonic cells grown under IND and PRE VBNC conditions using two different growth strategies: (i) planktonic cells grown for 24 h in 1% glucose and (ii) planktonic cells grown for 24 h in 0.4% glucose + 24 h in 1% glucose; in both strategies 20 mM of MgCl_2_ was added in the prevented condition. Data is represented as the result of the individual assays, where the horizontal lines represent the mean of at least three independent experiments. *p < 0.05, **p < 0.01, ***p < 0.001 (Unpaired Welch’s T-test). Glu, glucose.

After validating the applicability of the VBNC state induction model in biofilms formed by strain 1457, we became interested in determining if this experimental model was also applicable to planktonic cultures, something yet undetermined. As observed in biofilms, a significant decrease in the number of culturable cells was also found under VBNC inducing conditions for all isolates tested, whereas the total amount of planktonic cells was identical in both induced and prevented conditions (≈100%) (**Figure 3A**). Interestingly, for strain 1457, the induction of VBNC cells in planktonic cultures reached 48%, while in biofilms it reached 70%. However, these results are not directly comparable since growth conditions and incubation time differed: while VBNC cells induction in biofilms cultures was initiated in a pre-established 24 h-old biofilm, in planktonic cultures the VBNC state was induced from the start of the incubation period. Thus, to better mimic the biofilm experimental setup, another experiment was conducted, where planktonic cultures were first allowed to grow for 24 h, followed by another 24 h of growth in TSB with 1% glucose or 1% glucose plus 20 mM MgCl_2_. Notably, with longer incubation periods the proportion of VBNC cells in planktonic cultures (**Figure 3B**) reached similar levels to what was previously observed in biofilms (**Supplementary Figure 2**).

**Figure 4:**
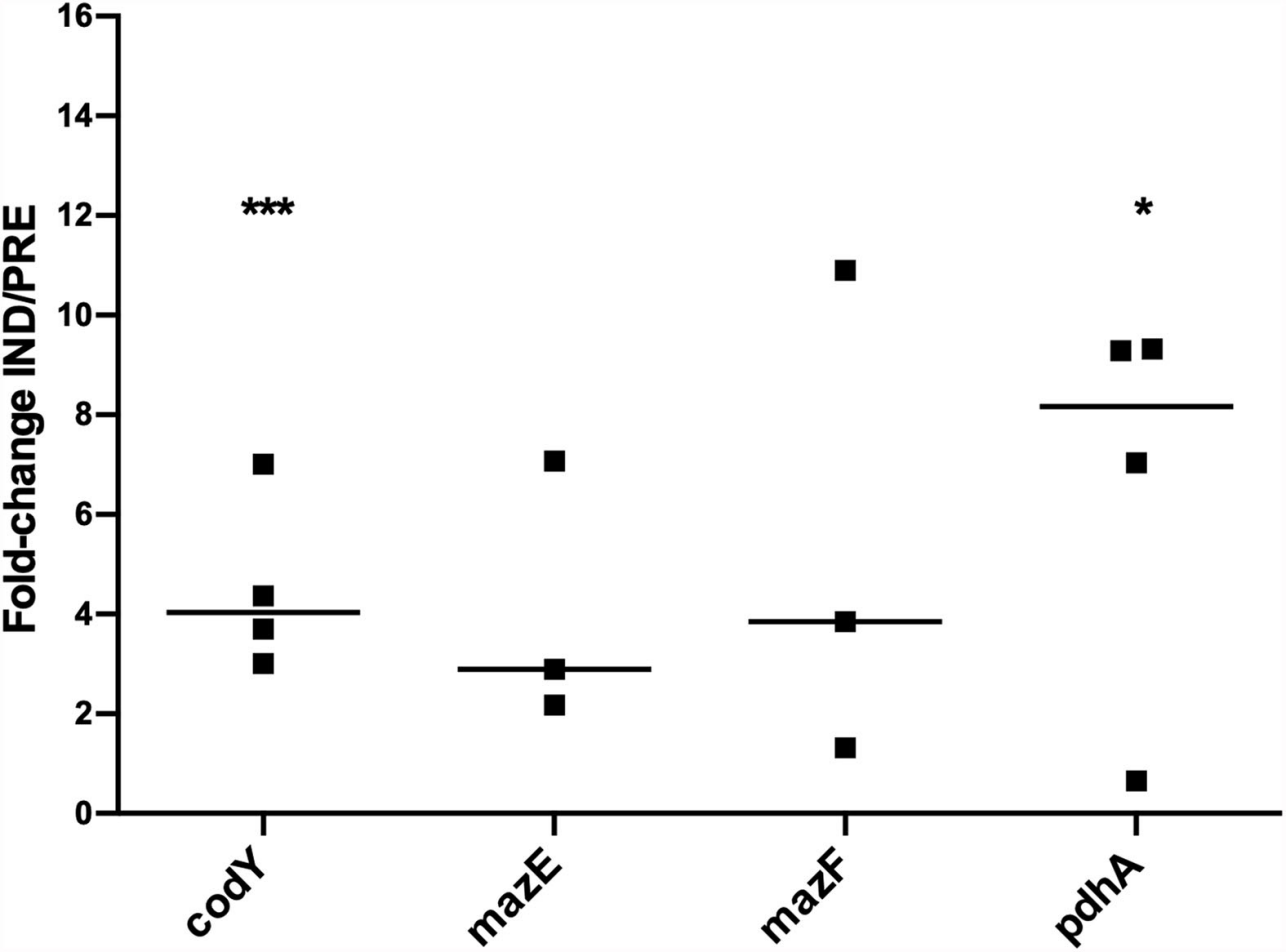
Expression of the genes of interest in 48 h-old planktonic populations of *S. epidermidis* 1457 grown under induced and prevented VBNC conditions. Data is presented as the fold-change between the induced (IND) and the prevented (PRE) VBNC conditions (IND/PRE). Data is represented as the fold-change of the individual assays, where the horizontal lines represent the median of at least three independent experiments. *p < 0.05, ***p < 0.001 (Unpaired Welch’s T-test).

Subsequently, we aimed to understand if the genes previously identified as potentially involved in the regulation of VBNC cells formation in biofilms could also play a role in planktonic cells. As can be seen in **Figure 4**, all genes were upregulated under the VBNC inducing conditions, with *codY* and *pdhA* reaching statistical significance. Interestingly, when comparing the fold-change IND/PREV between planktonic and biofilm cells (**Figures 2**, **4**), the fold-change IND/PRE of the *codY* and *pdhA* was higher in planktonic cells. Although the expression of both *mazE* and *mazF* genes seemed to be more pronounced when the VBNC state was induced in planktonic cultures, this difference was not statistically significant.

## Discussion

The induction of VBNC cells in *S. epidermidis* biofilms was previously reported in a wide range of clinical and commensal isolates, however, it was also found that not all strains formed VBNC cells under our *in vitro* VBNC state induction model (Cerca et al., 2011a; Carvalhais et al., 2018). Until now, the ability of *S. epidermidis* 1457, a strain widely used in genetic manipulation studies, to form VBNC cells using the previously developed model was unknown. Our results showed that the supplementation of the culture medium with 1% glucose induced a reduction in 1457 biofilm cells culturability of about 70% when compared to biofilms grown in media supplemented with 1% glucose plus 20 mM MgCl_2_.

Earlier, it was shown that the induction of VBNC cells in *S. epidermidis* biofilms led to modifications in cells transcriptomic and proteomic profiles. Carvalhais et al. reported, using an RNA-Seq approach, the upregulation of the genes *codY*, *mazE*, *mazF* and *pdhA* in *S. epidermidis* 9142 biofilms with higher proportions of VBNC cells, which indicated a potential involvement of these genes in the emergence of the VBNC state (Carvalhais et al., 2014). Even though RNA-Seq is a powerful technique to assess gene transcription, it is important to validate the results through an alternative method, being qPCR still considered the gold standard for gene expression quantification assays. Therefore, using qPCR, we first validated, in biofilm cultures, the RNA-Seq data previously obtained with strain 9142, and then assessed the expression of the genes of interest in strain 1457, as well as in the reference strains PT11004 and COM040A. Interestingly, using qPCR we were able to detect the expression of the gene *mazE* in both induced and prevented VBNC conditions, while in RNA-Seq analysis *mazE* was only detected in one of the conditions (Carvalhais et al., 2014). Additionally, *mazE* and *mazF* expression in strains 9142 and PT11004 was notably upregulated when the VBNC state was induced, but not in the target strain 1457, suggesting that *mazEF* might not have a key impact on VBNC state induction in this strain. On the contrary, *codY* and *pdhA* expression was noticeably upregulated in all strains tested, including 1457, which supports the hypothesis that these genes may be involved in the induction of VBNC cells. Unlike PdhA, the function of CodY has already been characterized in other strains. CodY is a repressor of hundreds of genes implicated in the transition from the exponential to the stationary growth phase, i.e., when nutrients become limited (Joseph et al., 2005; Barbieri et al., 2015). Additionally, it seems to regulate the *agr* quorum-sensing system, as well as genes associated with biofilm formation in *Staphylococcus aureus* (Majerczyk et al., 2008).

Although the onset of VBNC cells is commonly associated with the biofilm phenotype, non-culturable cells have also been reported in planktonic populations of several species, such as *S. aureus* and *Escherichia coli* (Xu et al., 2018). However, to our knowledge, no reports of VBNC cells in planktonic populations were found for *S. epidermidis.* As such, we aimed to understand if our VBNC state induction model could be applied to planktonic populations. As observed in biofilms, the decrease of culturability was more pronounced in the reference isolates, however, a significant proportion of VBNC cells was also obtained in planktonic populations of *S. epidermidis* 1457. Though, VBNC induction was significantly higher when planktonic populations were grown under biofilm-mimicking conditions (48 h of total growth), showing that the experimental setup with 24 h-old planktonic populations was not the most appropriate for the comparison with biofilms. Based on these results, we hypothesized that the lower proportion of VBNC cells in 24 h planktonic cultures may be, in part, related to the lower starting cell density, as in 24 h-old planktonic cultures the induction of the VBNC state started with ≈ 10^7^ CFU/mL, whereas in 48 h-old planktonic started with ≈ 10^9^ CFU/mL. The higher amount of VBNC cells attained in 48 h-old planktonic cultures raised our interest in understanding if the genes identified as playing a role in the emergence of VBNC cells in biofilms could also be involved in planktonic cultures. The analysis of gene expression showed that *codY* and *pdhA* were also upregulated in planktonic cultures under VBNC state inducing conditions. Although the exact function of *codY* and *pdhA* in the emergence of VBNC cells is still unknown, *codY* is responsible for the repression of genes when the cell is under unfavourable conditions, such as nutritional depletion and environmental stresses (Barbieri et al., 2015; Waters et al., 2016). This seems to be related to its upregulation when the VBNC condition is induced since the entrance of bacteria into a non-culturable state creates stress that leads to physiological and metabolic changes. On the other hand, although the role of *pdhA* has not yet been studied in *Staphylococcus* spp., the product of this gene – pyruvate dehydrogenase, has been related to the regulation of metabolism and perturbations on the cell membrane (Zhang et al., 2014; Singh et al., 2018).

Taken together, our data provide evidence that VBNC cells in *S. epidermidis* strain 1457 can be generated *in vitro*, both in biofilm and planktonic cultures. Additionally, this study reinforced the potential involvement of the genes *codY* and *pdhA* in VBNC cells formation in biofilm and revealed, for the first time, the potential involvement of these genes in VBNC cells formation in planktonic cultures. The role of both genes is now being assessed in our laboratory with knockout strains of *S. epidermidis* 1457.

## Data Availability Statement

The original contributions presented in the study are included in the article/**Supplementary Files**, further inquiries can be directed to the corresponding author/s.

## Author Contributions

Conceptualization, NC and AF. Investigation, VG and NL. Writing original draft, VG and NL. Writing—review and editing, NC and AF. Supervision, NC and AF. All authors contributed to the article and approved the submitted version.

## Funding

This work was supported by the Portuguese Foundation for Science and Technology (FCT) by the funded project PTDC/BIA-MOL/29553/2017, under the scope of COMPETE2020 (POCI-01-0145-FEDER-029553) and by the strategic funding of unit UIDB/04469/2020. VG and NL acknowledge the support of FCT individual fellowships [SFRH/BD/131452/2017 and SFRH/BD/136998/2018], respectively.

## Conflict of Interest

The authors declare that the research was conducted in the absence of any commercial or financial relationships that could be construed as a potential conflict of interest.

## Publisher’s Note

All claims expressed in this article are solely those of the authors and do not necessarily represent those of their affiliated organizations, or those of the publisher, the editors and the reviewers. Any product that may be evaluated in this article, or claim that may be made by its manufacturer, is not guaranteed or endorsed by the publisher.

## References

[B1] Barbieri G. Voigt B. Albrecht D. Hecker M. Albertini A. M. Sonenshein A. L.. (2015). CodY Regulates Expression of the Bacillus Subtilis Extracellular Proteases Vpr and Mpr. J. Bacteriol. 197, 1423–1432. doi: 10.1128/JB.02588-14 25666135 PMC4372738

[B2] Carvalhais V. França A. Cerca F. Vitorino R. Pier G. B. Vilanova M.. (2014). Dormancy Within Staphylococcus Epidermidis Biofilms: A Transcriptomic Analysis by RNA-Seq. Appl. Microbiol. Biotechnol. 98, 2585–2596. doi: 10.1007/s00253-014-5548-3 24504458

[B3] Carvalhais V. Pérez-Cabezas B. Oliveira C. Vitorino R. Vilanova M. Cerca N. (2018). Tetracycline and Rifampicin Induced a Viable But Nonculturable State in Staphylococcus Epidermidis Biofilms. Future Microbiol. 13, 27–36. doi: 10.2217/fmb-2017-0107 29227161

[B4] Cerca F. Andrade F. França A. Andrade E. B. Ribeiro A. Almeida A. A.. (2011a). *Staphylococcus Epidermidis* Biofilms With Higher Proportions of Dormant Bacteria Induce a Lower Activation of Murine Macrophages. J. Med. Microbiol. 60, 1717–1724. doi: 10.1099/jmm.0.031922-0 21799197 PMC10727147

[B5] Cerca F. França A. Perez-Cabezas B. Carvalhais V. Ribeiro A. Azeredo J.. (2014). Dormant Bacteria Within Staphylococcus Epidermidis Biofilms Have Low Inflammatory Properties and Maintain Tolerance to Vancomycin and Penicillin After Entering Planktonic Growth. J. Med. Microbiol. 63, 1274–1283. doi: 10.1099/jmm.0.073163-0 25053799 PMC4170483

[B6] Cerca F. Trigo G. Correia A. Cerca N. Azeredo J. Vilanova M. (2011b). SYBR Green as a Fluorescent Probe to Evaluate the Biofilm Physiological State of Staphylococcus Epidermidis, Using Flow Cytometry. Can. J. Microbiol. 57, 850–856. doi: 10.1139/W11-078 21950962

[B7] França A. Freitas A. I. Henriques A. F. Cerca N. (2012). Optimizing a qPCR Gene Expression Quantification Assay for S. Epidermidis Biofilms: A Comparison Between Commercial Kits and a Customized Protocol. PloS One 7, e37480. doi: 10.1371/journal.pone.0037480 22629403 PMC3357405

[B8] Freitas A. I. Vasconcelos C. Vilanova M. Cerca N. (2014). Optimization of an Automatic Counting System for the Quantification of Staphylococcus Epidermidis Cells in Biofilms. J. Basic Microbiol. 54, 750–757. doi: 10.1002/jobm.201200603 23686681

[B9] Galac M. R. Stam J. Maybank R. Hinkle M. Mack D. Rohde H.. (2017). Complete Genome Sequence of Staphylococcus Epidermidis 1457. Genome Announc, 5, e00450–17. doi: 10.1128/genomeA.00450-17 PMC5454206 28572323

[B10] Joseph P. Ratnayake-Lecamwasam M. Sonenshein A. L. (2005). A Region of *Bacillus Subtilis* CodY Protein Required for Interaction With DNA. J. Bacteriol. 187, 4127–4139. doi: 10.1128/JB.187.12.4127-4139.2005 15937175 PMC1151725

[B11] Keren I. Minami S. Rubin E. Lewis K. (2011). Characterization and Transcriptome Analysis of Mycobacterium Tuberculosis Persisters. MBio 2, 3–12. doi: 10.1128/mBio.00100-11 PMC3119538 21673191

[B12] Li Y. Huang T. Y. Mao Y. Chen Y. Shi F. Peng R.. (2020). Study on the Viable But non-Culturable (VBNC) State Formation of *Staphylococcus Aureus* and its Control in Food System. Front. Microbiol. 11, 599739. doi: 10.3389/fmicb.2020.599739 33324380 PMC7726111

[B13] Lleo M. M. Pierobon S. Tafi M. C. Signoretto C. Canepari P. (2000). mRNA Detection by Reverse Transcription-PCR for Monitoring Viability Over Time in an Enterococcus Faecalis Viable But Nonculturable Population Maintained in a Laboratory Microcosm. Appl. Environ. Microbiol. 66, 4564–4567. doi: 10.1128/AEM.66.10.4564-4567.2000 11010918 PMC92344

[B14] Mack D. Bartscht K. Fischer C. Rohde H. Grahl C. Dobinsky S.. (2001). Genetic and Biochemical Analysis of Staphylococcus Epidermidis Biofilm Accumulation. Methods Enzymol. 336, 215–239. doi: 10.1016/S0076-6879(01)36592-8 11398401

[B15] Mack D. Davies A. P. Harris L. G. Jeeves R. Pascoe B. Knobloch J. K.. (2013). “Staphylococcus Epidermidis in Biomaterial-Associated Infections,” in Biomaterials Associated Infection: Immunological Aspects and Antimicrobial Strategies. Eds. Moriarty F. Zaat S. A. J. Busscher H. J. (New York: Springer-Verlag), 25–56. doi: 10.1007/978-1-4614-1031-7

[B16] Mack D. Siemssen N. Laufs R. (1992). Parallel Induction by Glucose of Adherence and a Polysaccharide Antigen Specific for Plastic-Adherent Staphylococcus Epidermidis: Evidence for Functional Relation to Intercellular Adhesion. Infect. Immun. 60, 2048–2057. doi: 10.1128/iai.60.5.2048-2057.1992 1314224 PMC257114

[B17] Majerczyk C. D. Sadykov M. R. Luong T. T. Lee C. Somerville G. A. Sonenshein A. L. (2008). *Staphylococcus Aureus* CodY Negatively Regulates Virulence Gene Expression. J. Bacteriol. 190, 2257–2265. doi: 10.1128/JB.01545-07 18156263 PMC2293212

[B18] Otto M. (2009). *Staphylococcus Epidermidis* – The “Accidental” Pathogen. Nat. Rev. Microbiol. 7, 555–567. doi: 10.1038/nrmicro2182.Staphylococcus 19609257 PMC2807625

[B19] Postnikova O. A. Shao J. Mock N. M. Baker C. J. Nemchinov L. G. (2015). Gene Expression Profiling in Viable But Nonculturable (VBNC) Cells of *Pseudomonas Syringae* Pv. *Syringae* . Front. Microbiol. 6, 1419. doi: 10.3389/fmicb.2015.01419 26733964 PMC4683178

[B20] Rani S. A. Pitts B. Beyenal H. Veluchamy R. A. Lewandowski Z. Davison W. M.. (2007). Spatial Patterns of DNA Replication, Protein Synthesis, and Oxygen Concentration Within Bacterial Biofilms Reveal Diverse Physiological States. J. Bacteriol. 189, 4223–4233. doi: 10.1128/JB.00107-07 17337582 PMC1913414

[B21] Singh V. K. Sirobhushanam S. Ring R. P. Singh S. Gatto C. Wilkinson B. J. (2018). Roles of Pyruvate Dehydrogenase and Branched-Chain a-Keto Acid Dehydrogenase in Branched-Chain Membrane Fatty Acid Levels and Associated Functions in *Staphylococcus Aureus* . J. Med. Microbiol. 67, 570–578. doi: 10.1099/jmm.0.000707 29498620 PMC5982145

[B22] Sousa C. França A. Cerca N. (2014). Assessing and Reducing Sources of Gene Expression Variability in Staphylococcus Epidermidis Biofilms. Biotechniques 57, 295–301. doi: 10.2144/000114238 25495729

[B23] Untergasser A. Cutcutache I. Koressaar T. Ye J. Faircloth B. C. Remm M.. (2012). Primer3 - New Capabilities and Interfaces. Nucleic Acids Res 40, e115. doi: 10.1093/nar/gks596 22730293 PMC3424584

[B24] Waters N. R. Samuels D. J. Behera R. K. Livny J. Rhee K. Y. Sadykov M. R.. (2016). A Spectrum of CodY Activities Drives Metabolic Reorganization and Virulence Gene Expression in Staphylococcus Aureus. Mol. Microbiol. 101, 495–514. doi: 10.1111/mmi.13404 27116338 PMC5007081

[B25] Winstel V. Kühner P. Krismer B. Peschel A. Rohde H. (2015). Transfer of Plasmid DNA to Clinical Coagulase-Negative Staphylococcal Pathogens by Using a Unique Bacteriophage. Appl. Environ. Microbiol. 81, 2481–2488. doi: 10.1128/AEM.04190-14 25616805 PMC4357934

[B26] Winstel V. Kühner P. Rohde H. Peschel A. (2016). Genetic Engineering of Untransformable Coagulase-Negative Staphylococcal Pathogens. Nat. Protoc. 11, 949–959. doi: 10.1038/nprot.2016.058 27101516

[B27] Xu Z. Cheng C. Shen J. Lan Y. Hu S. Han W.. (2018). *In Vitro* Antimicrobial Effects and Mechanisms of Direct Current Air-Liquid Discharge Plasma on Planktonic *Staphylococcus Aureus* and *Escherichia Coli* in Liquids. Bioelectrochemistry 121, 125–134. doi: 10.1016/j.bioelechem.2018.01.012 29413862

[B28] Zandri G. Pasquaroli S. Vignaroli C. Talevi S. Manso E. Donelli G.. (2012). Detection of Viable But non-Culturable Staphylococci in Biofilms From Central Venous Catheters Negative on Standard Microbiological Assays. Clin. Microbiol. Infect. 18, E259–E261. doi: 10.1111/j.1469-0691.2012.03893.x 22578149

[B29] Zhang S. Guo L. Yang K. Zhang Y. Ye C. Chen S.. (2018). Induction of Escherichia Coli Into a VBNC State by Continuous-Flow UVC and Subsequent Changes in Metabolic Activity at the Single-Cell Level. Front. Microbiol. 9, 2243. doi: 10.3389/fmicb.2018.02243 30319570 PMC6167417

[B30] Zhang S. Hulver M. W. McMillan R. P. Cline M. A. Gilbert E. R. (2014). The Pivotal Role of Pyruvate Dehydrogenase Kinases in Metabolic Flexibility. Nutr. Metab. 11, 10. doi: 10.1186/1743-7075-11-10 PMC3925357 24520982

