# Supplementary material for: codY and pdhA Expression Is Induced in Staphylococcus epidermidis Biofilm and Planktonic Populations With Higher Proportions of Viable but Non-Culturable Cells
Source: Front Cell Infect Microbiol. 2021 Nov 15;11:771666. doi: 10.3389/fcimb.2021.771666 (PMC8634161; doi:10.3389/fcimb.2021.771666)
Supplement: Supplementary file 1 [file DataSheet_1.docx]

Supplementary Material

**Supplementary Table 1.** List of primers used for qPCR analysis

| **Target gene** | **Forward sequence**  **(5’-3’)** | **Reverse sequence**  **(3’-5’)** | **Amplicon**  **(bp)** | **Efficiency**  **(%)** |
| --- | --- | --- | --- | --- |
| *16S rRNA* | GGGCTACACACGTGCTACAA | GTACAAGACCCGGGAACGTA | 176 | 100.0 |
| *gyrB* | GCATTTGGTACGGGTATTGG | CATCAACATCGGCATCAGTC | 88 | 93.0 |
| *codY* | TTCGCCACCACCTAAAATTGG | AGAAGATCGACATATACCGCGTGA | 166 | 88.3 |
| *mazE* | CAAAATAGAAACCACAGTCTTGAAC | AGATATTAAATGTGATTCATTGCAATC | 138 | 91.8 |
| *mazF* | GAAGAGGAGATGTTTATTTAGCGG | CCCAAACTAATATCTAAGGCATTATC | 325 | 95.5 |
| *pdhA* | GCTTCTTGTCCTGCTGTTGGTG | TTGGATGAAGACGGAAATGTCG | 177 | 99.4 |


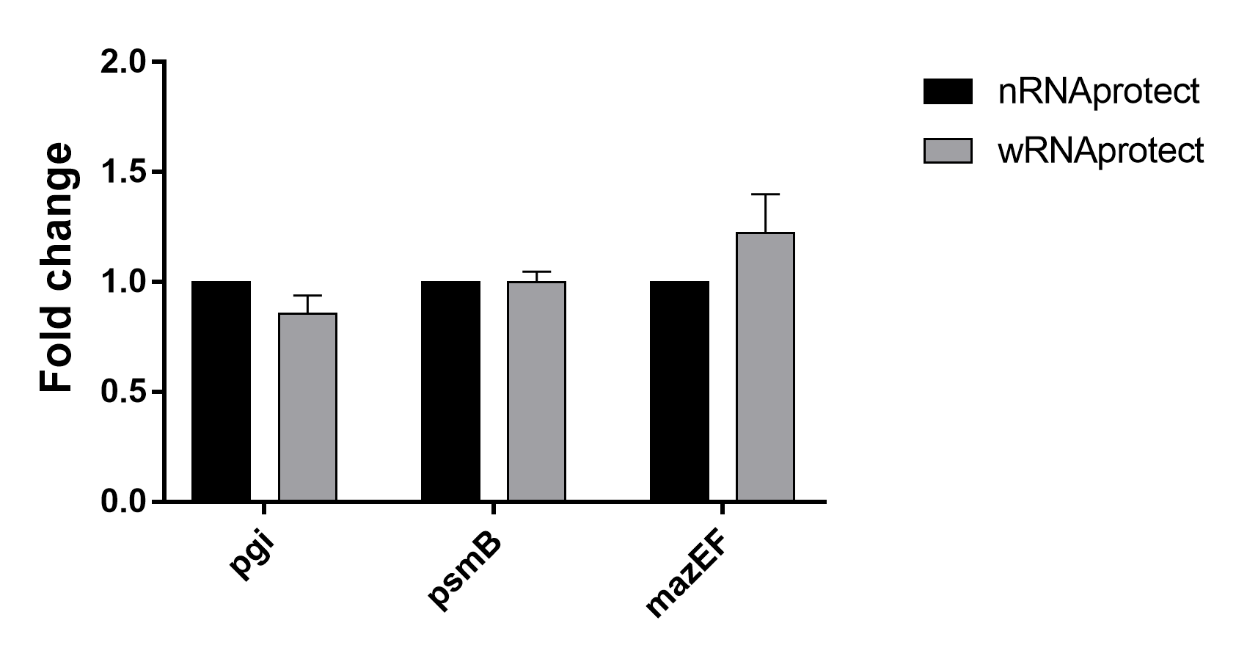


**Supplementary Figure 1.** Gene expression after immediate RNA isolation in the absence or presence of RNA protect bacteria reagent. The bars represent the average of three technical qPCR replicates plus SEM, completed with RNA from one isolation. Information about Glucose-6-phosphate isomerase (*pgi*) and phenol soluble modulin beta (*psmB*) primers can be seen in (Carvalhais et al., 2013). nRNAprotect, no RNA protect; wRNAprotect, with RNA protect.


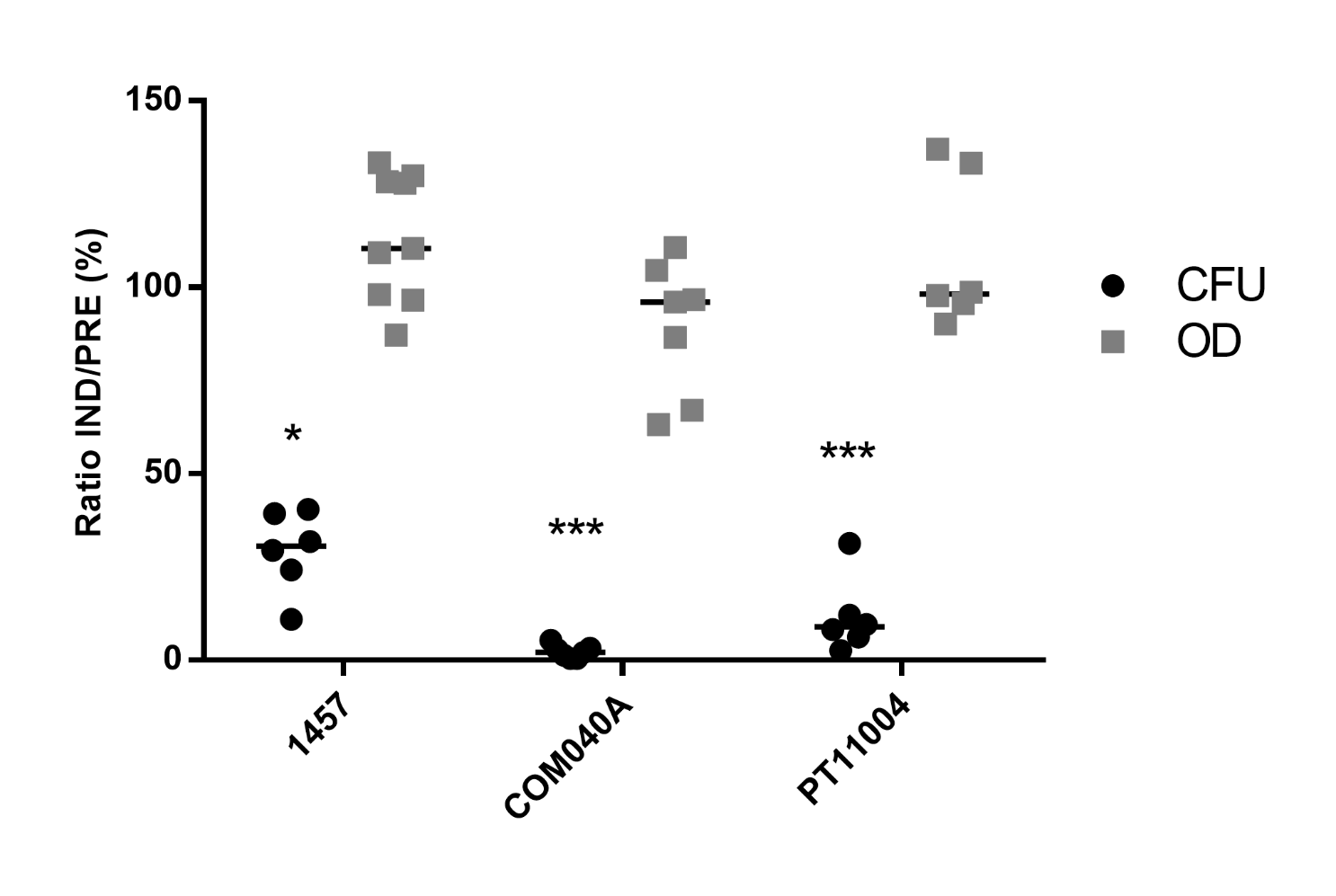


**Supplementary Figure 2.** Culturability (CFU/mL) and total amount of cells (OD_640nm_) of *S. epidermidis*  1457, COM040A and PT11004 48 h-old biofilms grown under induced (IND) or prevented (PRE) VBNC conditions. Data is represented as the ratio (%) of the culturability (CFU) or OD between cells in the IND or PRE VBNC state. The results are displayed as the mean + standard deviation of six independent experiments. **p*<0.05, ****p*<0.001(Unpaired Welch’s T-test).

**References**

Carvalhais, V., Delgado-Rastrollo, M., Melo, L.D.R., Cerca, N. (2013) Controlled RNA contamination and degradation and its impact on qPCR gene expression in *S. epidermidis* biofilms. *J Microbiol Methods* 95,195–200. doi:10.1016/j.mimet.2013.08.010.
